# Genetic targeting of B-Raf^V600E^ affects survival and proliferation and identifies selective agents against *BRAF*-mutant colorectal cancer cells

**DOI:** 10.1186/1476-4598-13-122

**Published:** 2014-05-24

**Authors:** Benjamin Hirschi, Eike Gallmeier, Andreas Ziesch, Maximilian Marschall, Frank T Kolligs

**Affiliations:** 1 Department of Internal Medicine II, University of Munich, Marchioninistr. 15, D-81377 Munich, Germany; 2 German Cancer Consortium (DKTK), German Cancer Research Center (DKFZ), Heidelberg, Germany; 3 Current address: Institute of Stem Cell Research, Helmholtz Center Munich, Ingolstädter Landstr. 1, 85764 Neuherberg, Germany

**Keywords:** BRAF, Colorectal cancer, Knockout, Pharmacogenetics

## Abstract

**Background:**

Colorectal cancers carrying the B-Raf V600E-mutation are associated with a poor prognosis. The purpose of this study was to identify B-Raf^V600E^-mediated traits of cancer cells in a genetic *in vitro* model and to assess the selective sensitization of B-Raf^V600E^-mutant cancer cells towards therapeutic agents.

**Methods:**

Somatic cell gene targeting was used to generate subclones of the colorectal cancer cell line RKO containing either wild-type or V600E-mutant B-Raf kinase. Cell-biologic analyses were performed in order to link cancer cell traits to the *BRAF*-mutant genotype. Subsequently, the corresponding tumor cell clones were characterized pharmacogenetically to identify therapeutic agents exhibiting selective sensitivity in B-Raf^V600E^-mutant cells.

**Results:**

Genetic targeting of mutant *BRAF* resulted in restoration of sensitivity to serum starvation-induced apoptosis and efficiently inhibited cell proliferation in the absence of growth factors. Among tested agents, the B-Raf inhibitor dabrafenib was found to induce a strong V600E-dependent shift in cell viability. In contrast, no differential sensitizing effect was observed for conventional chemotherapeutic agents (mitomycin C, oxaliplatin, paclitaxel, etoposide, 5-fluorouracil), nor for the targeted agents cetuximab, sorafenib, vemurafenib, RAF265, or for inhibition of PI3 kinase. Treatment with dabrafenib efficiently inhibited phosphorylation of the B-Raf downstream targets Mek 1/2 and Erk 1/2.

**Conclusion:**

Mutant *BRAF* alleles mediate self-sufficiency of growth signals and serum starvation-induced resistance to apoptosis. Targeting of the *BRAF* mutation leads to a loss of these hallmarks of cancer. Dabrafenib selectively inhibits cell viability in B-Raf^V600E^ mutant cancer cells.

## Background

Colorectal cancer (CRC) is one of the most frequent causes of cancer related morbidity and mortality [1]. In advanced stages of colorectal cancer, individualized tumor therapy with molecularly targeted agents has been introduced into clinical practice. The antibody cetuximab, which is directed against the epidermal growth factor receptor (EGFR), provides survival advantage in the subgroup of patients carrying wild type KRAS alleles [2]. The *KRAS* mutational status is predictive in terms of response to therapy with antibodies targeting the EGFR.

In CRC, *BRAF* is mutated with a prevalence of 9.6% [3] and the T1799A mutation accounts for more than 80% of these mutation events, resulting in a hyperactivating substitution of valine^600^ by glutamic acid [4]. CRC patients with tumors harboring the B-Raf V600E mutation have a poor prognosis [2]. The mutant kinase constitutively activates the mitogen activated cascade of the mitogen-activated protein kinase (MAPK) pathway, resulting in deregulation of MAPK target genes. In addition to the pleiotropic functions of the MAPK pathway, the mammalian target of rapamycin (mTOR) pathway is likewise affected due to crosstalk via extracellular signal regulated kinase (Erk) [5]. Furthermore, the B-Raf V600E mutation is associated with a scope of cellular phenotypes, including resistance to apoptosis, genetic instability, senescence, and complex mechanisms providing independence from extracellular growth signals [6].

For this study, we established an *in vitro BRAF* model system ideally suited for pharmacogenetic analyses by recombination of either V600E or wild-type *BRAF* in the colorectal cancer cell line RKO. RKO exhibits all key traits of a distinct subpopulation of colorectal cancer patients, namely V600E mutant B-Raf, microsatellite instability (MSI), and the CpG island methylator phenotype (CIMP) [7-9]. In addition, since RKO is wild-type for *KRAS*, *APC*, and *TP53*, and lacks chromosomal instability (CIN), all relevant molecular features of other CRC subtypes are missing in these cells [10-13]. We used this model system to study cancer cells traits depending on B-Raf^V600E^ and to identify agents selectively targeting *BRAF*-mutant cells.

## Results and discussion

### *BRAF* targeting in RKO

**Figure 1:**
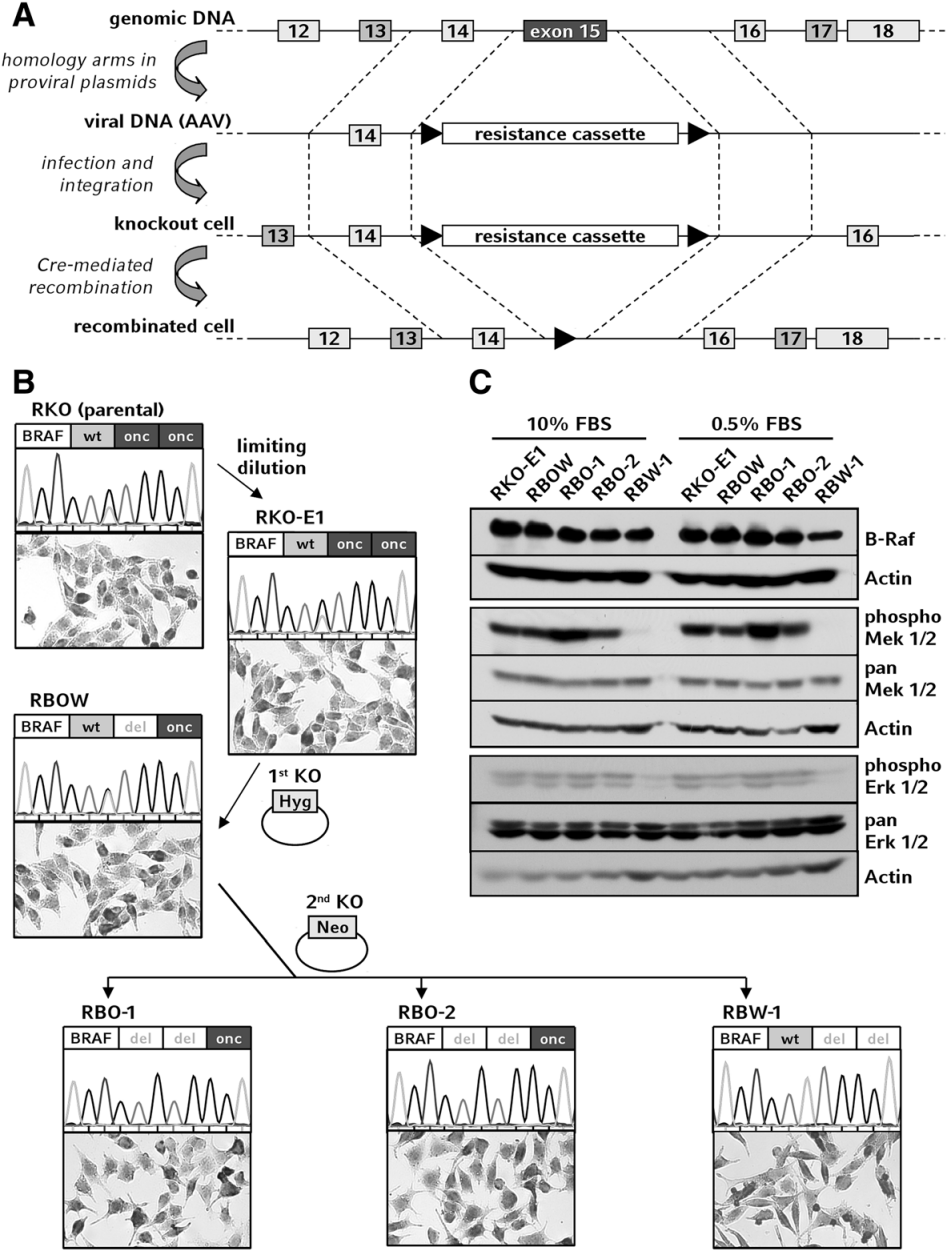
**Generation and validation of *BRAF *knockout cell lines. A:** Schematic representation of the knockout procedure resulting in recombination of *BRAF* exon 15 and substitution by a resistance cassette. **B:** Genealogy of the corresponding tumor cell clones. From the parental colorectal cancer cell line RKO a single clone was generated by limiting dilution. Subsequently, a first oncogenically mutant allele (onc) was deleted by infection with AAV-BRAF-Hyg virus and the cell line RBOW (RKO-derived clone *BRAF*^-/onc/wt^) was established. In a secondary targeting using AAV-BRAF-Neo virus either the second V600E allele or the wild-type allele (wt) was targeted to generate RBO (RKO-derived clone *BRAF*^-/-/onc^) and RBW (RKO-derived clone *BRAF*^-/-/wt^) clones. Genotypes were validated by A/T peak ratio in sequencing histograms. Microscopy scales: 250 μm × 150 μm. **C:** Aliquots of RKO-E1 and knockout cell clones were incubated under different serum conditions, subsequently lyzed, and used for Western blot analysis.

It has been shown that B-Raf^V600E^ is sufficient to promote proliferation via Erk 1/2 signaling independently of exogenous growth factors and confers mechanisms to evade apoptosis [14-16]. However, these results are primarily based on non-quantitative RNA interference (RNAi) methods which are prone to artifacts in mammalian cells due to nonspecific defense mechanisms [17]. In contrast, somatic cell gene targeting enables quantitative knockouts of single alleles (Figure 1A) and the generation of endogenous models featuring well-defined genetic backgrounds [18]. Utilizing this method, we have disrupted *BRAF* alleles in the colorectal cancer cell line RKO and established syngeneic clones which harbor a single *BRAF* allele of either wild-type or mutant genotype. Despite its near-diploid karyotype and MSI phenotype, the colorectal cancer cell line RKO carries a stable triplication of the *BRAF* gene locus (dup (7) (q21q36)) with one wild-type and two mutant alleles present in parental cells [13]. This genotype was verified by DNA sequencing in RKO-E1, a subclone obtained from RKO that was found to be comparable to the parental cell line in terms of morphology and proliferation (Figure 1B and data not shown).

In the first targeting round, an oncogenic allele of *BRAF* exon 15 was recombined and deleted by somatic cell gene targeting to generate the cell clone RBOW (RKO-derived *BRAF*^onc/wt/-^). Subsequently, either wild-type or V600E-mutant B-Raf was disrupted by targeting a second allele in RBOW, yielding six *BRAF*-mutant and one wild-type clone from approximately 10^4^ screened colonies. Out of these double positive clones, *BRAF* knockout cell lines RBO-1 and RBO-2 (RKO-derived *BRAF*^onc/-/-^ 1 and 2) as well as RBW-1 (RKO-derived *BRAF*^wt/-/-^) were established (Figure 1B). The apparent counterselection against inactivation of B Raf^V600E^ might indicate the presence of an oncogene addiction for B-Raf^V600E^ as a cancer cell trait in RKO [19].

For structural confirmation of the deleted alleles, DNA sequencing was performed and all genotypes were verified (Figure 1B). Furthermore, all cells expressed *BRAF* protein at comparable levels (Figure 1C). While the expression of Mek 1/2 and Erk 1/2 was independent of serum concentration and *BRAF* status, the phosphorylation of these effector kinases was constantly active in the *BRAF*-mutant clones but low in *BRAF*-wild-type cells (Figure 1C). This was found to be independent of the serum concentration, indicating that the phosphorylation status of Mek and Erk is dependent on mutant *BRAF* in RKO.

### Cell-biological phenotypes related to mutant *BRAF*

**Figure 2:**
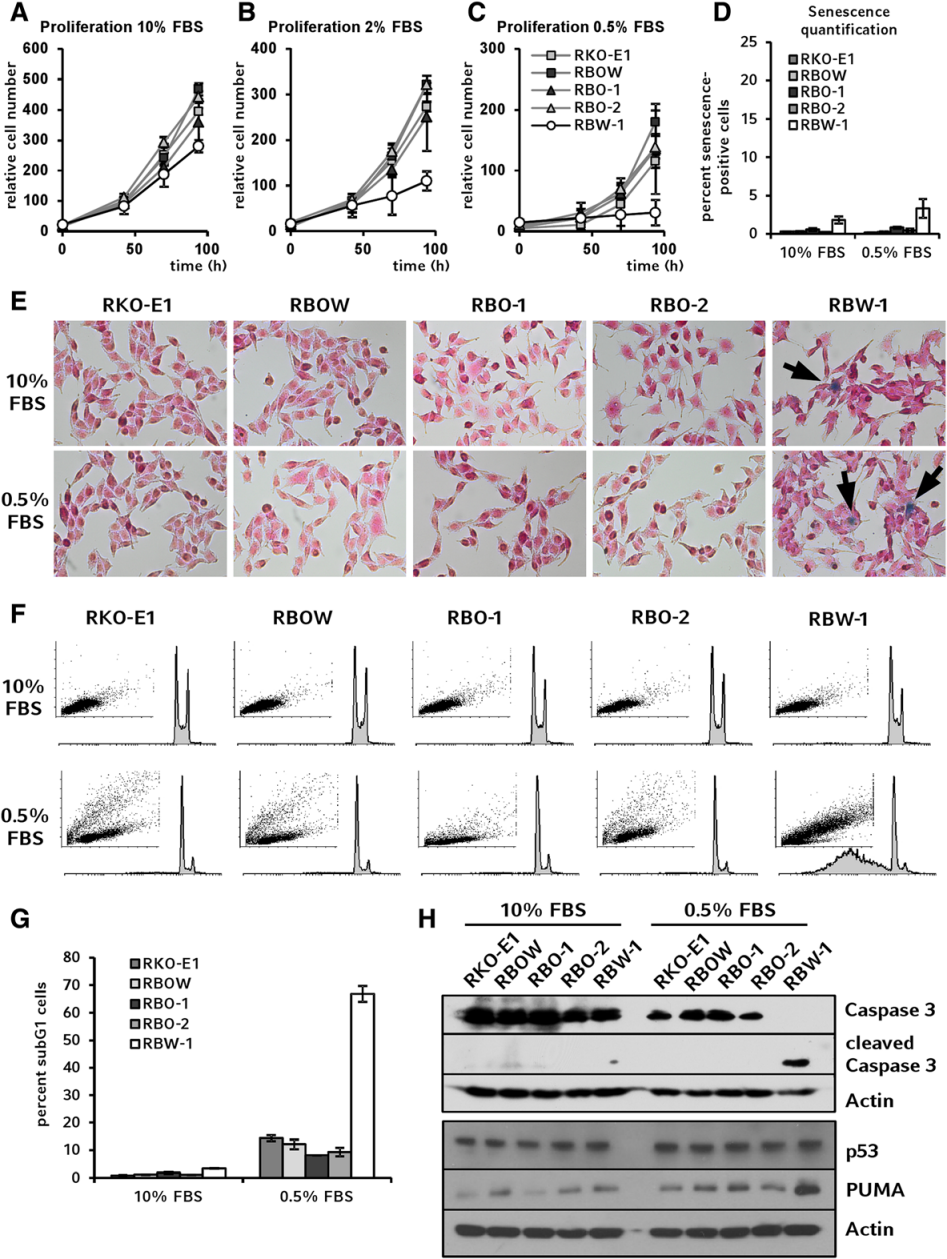
**Characterization of proliferation behavior. A-C**: Proliferation rates of cells as assessed by manual counting. **D-E**: Staining of senescence-associated β-galactosidase activity and quantification of senescent cells revealed no relevant amounts of cellular senescence to be present in RBW-1 or other clones. Microscopy scale: 150 μm × 100 μm. **F**: In 10% FBS all cell lines showed similar cell cycle patterns in flow cytometry. Reduction of serum led to aberrant patterns for RBW-1 compared to *BRAF*-mutant clones. **G**: Quantification of sub G1 fraction revealed high amounts of cell death in RBW-1 after incubation with 0.5% FBS. **H**: Apoptosis was analyzed by Western blotting for cleaved caspase 3, p53, and PUMA.

Under standard long-term cell culture conditions no differences in morphology or growth were observed between the cell clones (Figures 1B and 2A). Expectedly, decreased serum concentrations led to lower proliferation rates in these cells, but exponential growth was sustained under all applied conditions. However, the withdrawal of serum resulted in the inhibition of cell growth of the wild-type cells RBW-1 (Figure 2B and C).

It has been shown previously that *BRAF* wild-type cells require glucose supply for survival whereas *BRAF*-mutant cell clones maintain proliferation in low-glucose environments [20]. Here we show that the V600E mutation of B-Raf also provides independency of serum-derived growth signals in RKO and that targeting of oncogenically mutant *BRAF* is sufficient to deprive this vital feature of malignancy from the cells, thereby corroborating previous reports [6]. Sustained proliferative signaling is considered one of the major traits of cancer cells and is therefore used as a target mechanism of individualized therapy approaches including anti EGFR therapy strategies in colorectal cancer [21,22].

In another context, mutant B-Raf induced cellular senescence rather than proliferation [23,24]. However, senescence can be overcome by phosphoinositide 3-kinase (PI3K)/AKT signaling [24] which is hyperactivated in RKO due to a *PIK3CA* mutation. By staining of senescence-associated β-galactosidase activity [25] we examined whether the differential proliferation rates observed upon serum deprivation were attributable to cellular senescence. Cellular senescence was detected at very low levels in less than 5% of cells (Figure 2D-E), indicating that senescence alone cannot explain the strong reduction in cell growth observed upon withdrawal of serum.

Flow cytometry revealed a significant increase of apoptotic cells in wild-type compared to mutant clones upon withdrawal of serum (Figure 2F and G). Apoptosis was confirmed by the detection of cleaved caspase 3 at considerable levels in serum-starved RBW-1, while all other samples showed full-length protein only (Figure 2H). Consistent with RKO modeling a distinct subpopulation of patients characterized by the presence of certain molecular features and the absence of others [7], no implication of p53 in apoptosis was observed (Figure 2H). Since serum starvation is often used to model apoptosis mediated via the PUMA pathway [26], we also analyzed PUMA protein levels. PUMA was found to be highly abundant specifically in serum starved RBW-1 (Figure 2H). Consistent with data previously shown by others, starvation-induced apoptosis is mediated by PUMA in a p53-independent fashion in our experiments [27].

Programmed cell death is a key feature of proliferation control in homeostasis and overcoming apoptosis is considered another hallmark of cancer cells [28]. Since virtually all malignant cancer cells show apoptosis resistance, the induction of apoptotic pathways is considered a particularly promising approach for therapeutic strategies [29]. Our results show that in RKO this particular cancer cell trait is modulated by and dependent on B-Raf^V600E^ and that targeting mutant *BRAF* is sufficient to restore sensitivity to caspase-dependent apoptosis after serum withdrawal via p53-independent PUMA induction [27]. Complementing and extending previous studies, we thus provide evidence from an endogenous and quantitative genetic model of *BRAF*-mutant colorectal cancer cells, thereby ruling out the occurrence of artifacts caused by unspecific cellular response or incomplete knockdown in RNAi setups and, likewise, avoiding inter-species bias potentially experienced in mouse models of colorectal cancer [30].

### Pharmacogenetic characterization

**Figure 3:**
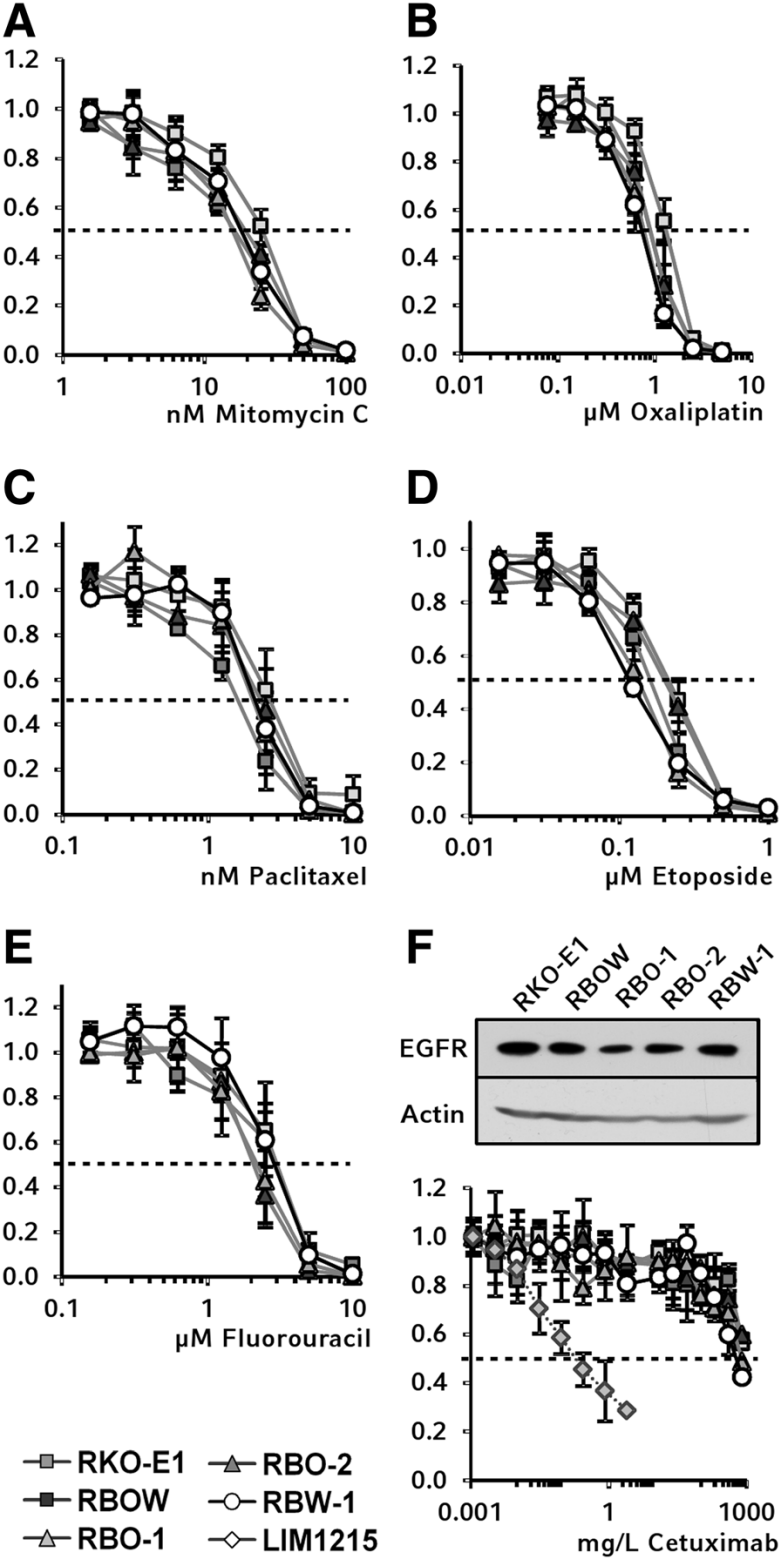
**Pharmacogenetic characterization of *BRAF *knockout cell clones.** Cells were incubated with different chemotherapeutic agents. On day 7 the cells were lyzed and proliferation was determined by SYBR green I staining of DNA. **A-E**: No differences in response were observed between *BRAF*-mutant and wild-type clones with mitomycin C, oxaliplatin, paclitaxel, etoposide, or 5-fluorouracil. **F**: EGFR expression was verified in all cell clones by Western blotting. No different proliferation behavior between the corresponding cell clones was observed upon treatment with monoclonal EGFR antibody cetuximab in concentrations up to 800 mg/L. As a positive control, the colorectal cancer cell line Lim1215 was used.

Hyperactivated Raf/Mek/Erk signaling has been suggested to mediate resistance towards drug-induced cell death [31,32]. However, data from prostate cancer cells transfected with mutant *BRAF* showed that there might be tumor entity-dependent differences [33]. Our model system of corresponding tumor cells is ideally suited to determine the B-Raf^V600E^-specific effects of a comprehensive panel of widely used chemotherapeutic agents including crosslinking agents (oxaliplatin, mitomycin C), a taxane (paclitaxel), a topoisomerase II inhibitor (etoposide), and the nucleic acid metabolism inhibitor 5-fluorouracil. We found that the *BRAF* mutational status did not have a detectable impact on chemosensitivity towards any of these agents (Figure 3A-E). These findings suggest that B-Raf^V600E^ does not significantly contribute to resistance towards conventional chemotherapeutics in colorectal cancer cells and are in accordance with previous studies suggesting the Raf/Mek/Erk cascade to play a minor role in chemoresistance [34,35]. Taken together with the observed differential sensitivity of *BRAF-*mutant cells towards starvation-induced apoptosis, these results further dissect the distinct apoptosis pathways in our model, i.e. serum-starvation versus chemotherapeutic agents.

The predictive role of *BRAF* mutations in EGFR antibody therapy has been elucidated recently but remains poorly understood on the molecular level [2]. Our model enabled us to specifically analyze *BRAF*-dependent effects of cetuximab sensitivity independent of confounding genetic events. RKO cells and derived mutant and wild type clones express the epidermal growth factor receptor (EGFR) at comparable levels (Figure 3F). To test whether loss of mutant *BRAF* might reconstitute responsiveness to the inhibition of EGFR, cells were treated with the monoclonal antibody cetuximab. However, no difference in proliferation was observed between *BRAF* wild-type and mutant cells, while cetuximab sufficiently inhibited growth of the control cell line Lim1215 [36]. All cells revealed a similar slight decrease in the proliferation index down to 0.6 at very high concentrations of cetuximab (Figure 3F). This modest effect might be due to unspecific toxicity or to dilution, rather than to a specific anti-proliferative effect of cetuximab, since at 0.8 g/L the antibody solution accounts for 16% of the culture medium. These findings are in line with previous studies showing that resistance against EGFR-targeted treatment frequently occurs in *BRAF*-mutant tumors [37].

**Figure 4:**
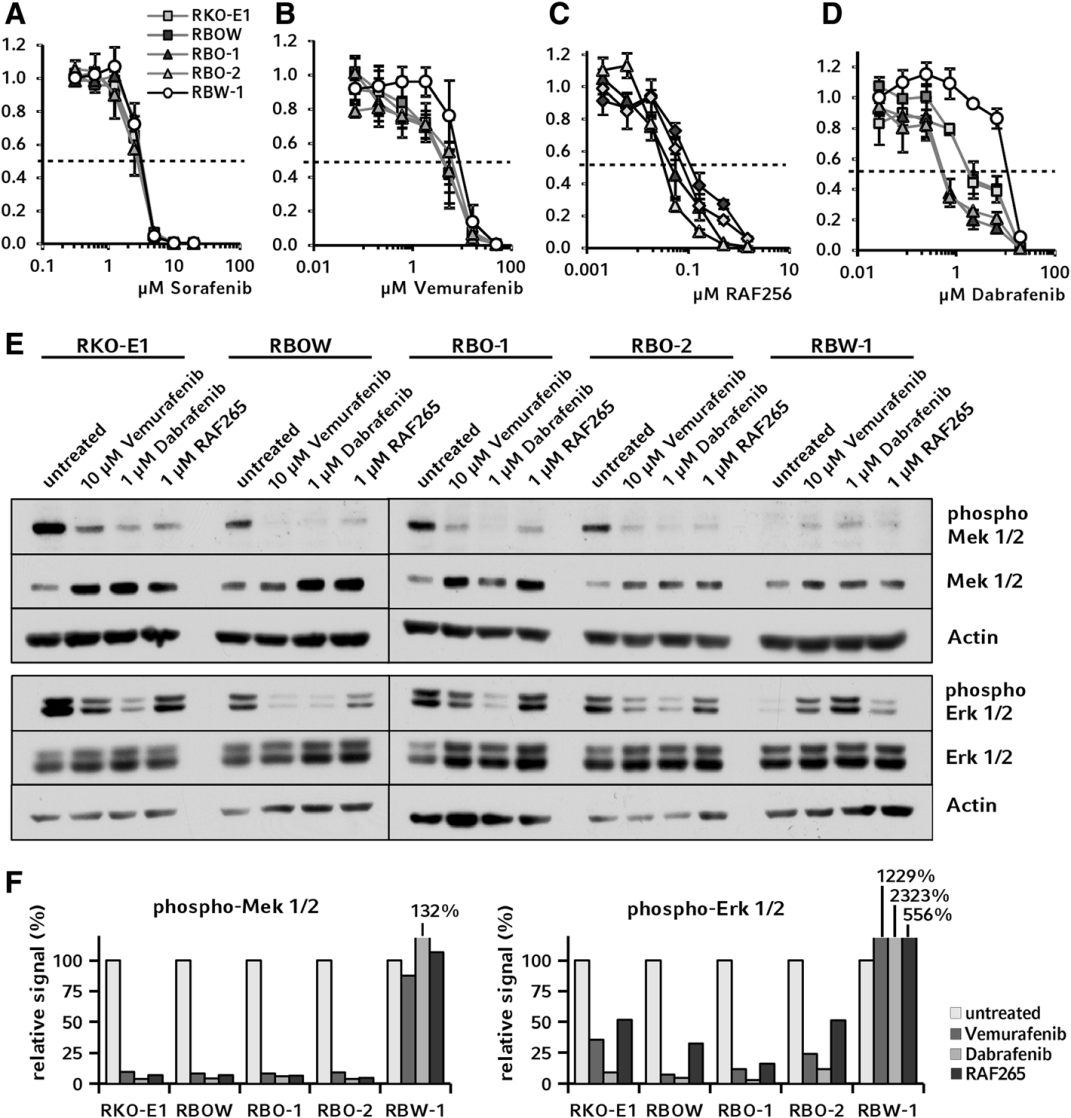
**Chemosensitivity for B-Raf inhibitors. A**: Multi-kinase inhibitor sorafenib had no differential effect on RKO *BRAF* knockout cell clones. **B-C**: Although no significant differences in the IC_50_ were observed, by trend RBW-1 was less sensitive to vemurafenib and RAF265 compared to cells with mutant *BRAF* alleles. **D**: The IC_50_ of the B-Raf kinase inhibitor dabrafenib in RBW-1 was 5.3 times higher than the IC_50_ of the parental clones RKO-E1 and RBOW, and 20 times higher than in clones carrying a *BRAF*-mutant allele only. **E**: Aliquots of each cell line were incubated with the respective IC_50_ concentrations of B-Raf inhibitors, lyzed, and analyzed in Western blots for phosphorylation of Mek 1/2 (upper panel) and Erk 1/2 (lower panel). **F**: Signal intensities of phospho-Mek 1/2 and phosphor-Erk 1/2 were normalized to the total fractions in densitometric analyses.

Next, we investigated the impact of the *BRAF* V600E-mutation on several established B-Raf inhibitors. Sorafenib was developed as the first small molecule inhibitor selectively targeting Raf kinases and has been reported to inhibit B-Raf [38,39]. However, sorafenib was found to show a complex inhibition profile affecting various effector kinases in several cellular signaling pathways and is therefore considered a multi-kinase inhibitor today [40]. Recently, it has been shown that PI3K/AKT signaling rather than the Raf/Mek/Erk cascade is both the main target of sorafenib in apoptosis initiation and a key player in *de novo* resistance against sorafenib [41,42]. In our model, sorafenib suppressed proliferation at anticipated concentrations, but elicited no differential effects between *BRAF*-mutant and wild-type cells (Figure 4A). This further supports the mechanism of sorafenib to be widely independent of Mek 1/2 phosphorylation.

Recently, more selective B-Raf inhibitors have been developed exhibiting considerable specificity for the V600E mutant kinase *in vitro*[43,44]. Testing these compounds in our model system revealed that vemurafenib and RAF265 did not have significantly different effects on proliferation of the RKO-derived clones (Figure 4B and C). Mechanisms of resistance against B-Raf inhibition are complex and involve activation of upstream rather than only downstream effectors of the B-Raf kinase or can be modulated via other signaling pathways [45,46]. Additionally, resistance against particular B-Raf inhibitors has recently been reported to occur frequently in colorectal cancer cells [47,48].

In contrast to these compounds, the B-Raf^V600E^ inhibitor dabrafenib selectively decreased proliferation of *BRAF*-mutant cell clones (Figure 4D). Remarkably, the IC_50_ ratio between the wild-type clone RBW-1 and clones carrying a mutant allele only (RBO-1, RBO-2) was 20, while it was only 5.3 between RBW-1 and the heterozygous clones (RKO-E1, RBOW) that carry both wild-type and mutant alleles, potentially indicating a gene-dosage effect [49]. However, since the inhibition profile of dabrafenib is not yet fully known, a favorable off-target effect cannot be excluded and should be further examined in future studies.

To further investigate the differential effects of the specific B-Raf^V600E^ inhibitors, we examined their specific impact on downstream effectors of B-Raf. For this purpose, we analyzed the relative phosphorylation levels of Mek 1/2 and Erk 1/2 in lysates from cells incubated with compound concentrations corresponding to the previously determined IC_50_ (Figure 4E upper panel and Figure 4F left panel). All inhibitors reduced the relative level of Mek 1/2 phosphorylation in clones carrying the V600E mutation by more than 90% with dabrafenib showing the strongest effect. No reduction of Mek 1/2 phosphorylation was observed in RBW-1 *BRAF*^wt/-/-^ cells. These data were further confirmed on the level of phospho-Erk 1/2 (Figure 4E lower panel and Figure 4F right panel). Taken together, analysis of B-Raf downstream signaling showed dabrafenib to inhibit the Raf/Mek/Erk cascade most efficiently. In RBW-1 cells, a paradoxical elevation of phosphorylated Mek 1/2 and Erk 1/2 levels was observed upon B-Raf inhibition, a phenomenon previously reported for *BRAF* wild-type cells [50-52].

### PI3K/AKT signaling in corresponding cell clones

Although the MAPK signaling and PI3K/AKT signaling pathways feature multiple interconnections, they are commonly considered as two distinct pathways [53]. Sharing EGFR as an activating upstream growth factor receptor, the MAPK and PI3K/AKT axes mediate different cellular outcomes by complex temporal phosphorylation patterns, rather than by exclusive activation of a single cascade [54]. The parental RKO cells harbor prominent mutations in both axes of this signaling network, namely B-Raf^V600E^ and p110α^H1047R^. Therefore, the corresponding knockout clones were tested for differential sensitivity towards inhibition of the PI3K/AKT axis.

**Figure 5:**
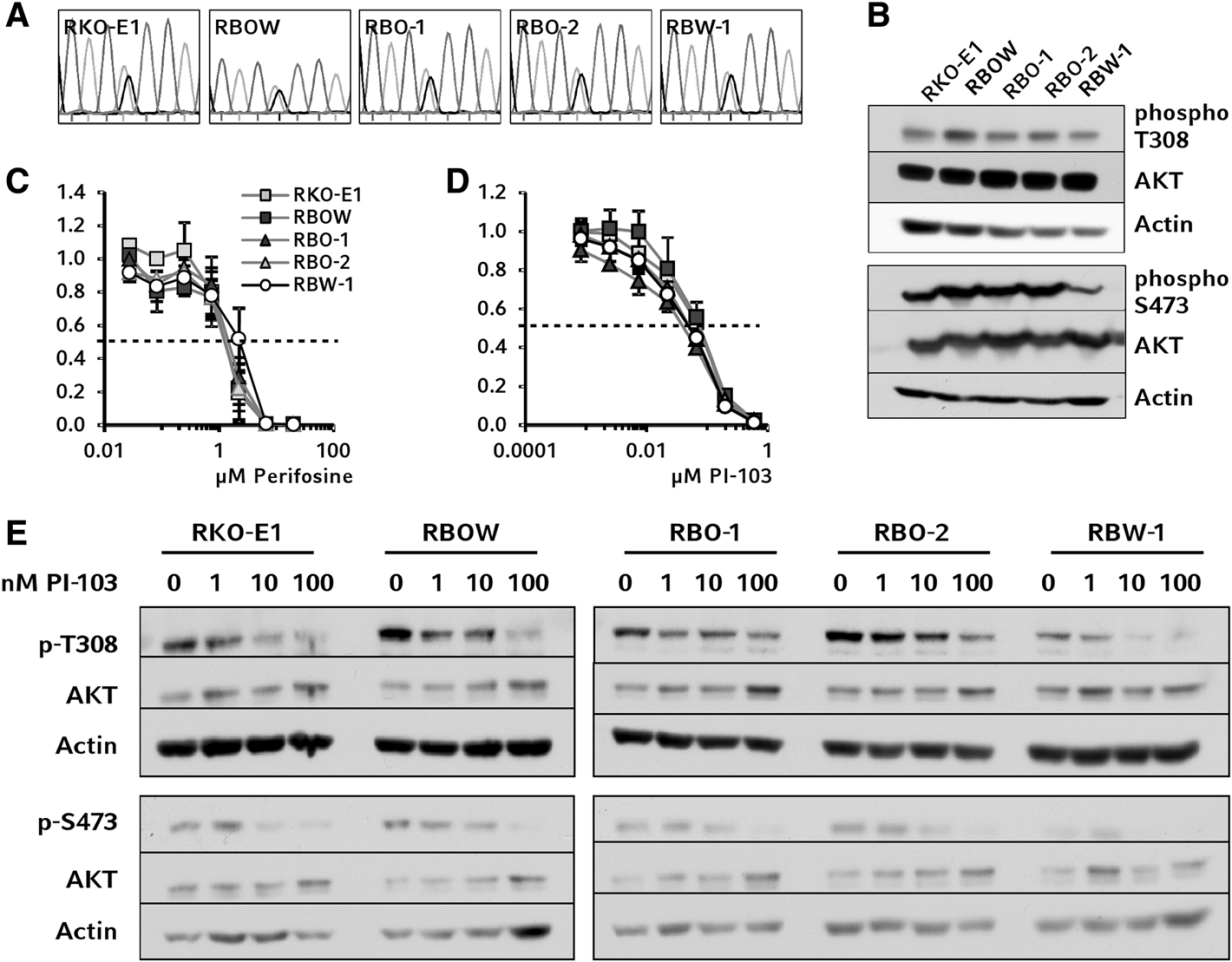
**Analysis of PI3K/AKT signaling. A**: Sequencing results for *PIK3CA* A3140G corresponding to the H1047R mutation in the catalytic subunit of PI3 kinase are shown as histograms. **B**: Phospho-protein and total protein levels for AKT were examined by Western Blotting. **C-D**: No differential response between wild-type and *BRAF*-mutant cell clones was found in proliferation assays. **E**: In Western blot analysis, PI-103 inhibited phosphorylation of AKT residues T-308 and S-473 in a dose-dependent manner in all cell clones.

A heterozygous mutation of *PIK3CA* was confirmed in all RKO-derived cell clones (Figure 5A). Without treatment, phosphorylation of AKT was decreased in *BRAF* wild-type cells at both T-308 and at S-473, with the effects on S-473 being more pronounced (Figure 5B). Upon treatment with perifosine, an inhibitor of both Erk 1/2 and AKT kinases, no differential sensitivity was observed for *BRAF* wild-type cells (Figure 5C). Next, the cells were treated with an inhibitor of the PI3K catalytic subunit, PI-103, as a more upstream-acting agent. Again, no differential sensitivity was observed between *BRAF*-mutant and wild-type clones (Figure 5D).

In Western blot analyses, no decrease in AKT phosphorylation was observed upon treatment with perifosine at IC_75_ for any of the cell clones (data not shown). This likely indicates the consistent decrease in proliferation of the cell clones to be caused by unspecific cell toxicity of the compound. However, western blot analysis revealed a robust inhibition of AKT phosphorylation at any applied concentration of PI-103. Even in wild-type cells, which showed lower phospho-AKT levels as compared to mutant cells under standard conditions, phosphorylation of AKT was further decreased upon PI-103 treatment (Figure 5E).

Combined targeting of MAPK signaling and PI3K/AKT signaling is considered a promising therapeutic strategy for tumor cells. Consistently, a combinatorial approach has recently been shown to synergistically inhibit proliferation in RKO cells [55]. While the relatively high concentration of vemurafenib needed to inhibit cell proliferation was confirmed in our model, both *BRAF* wild-type and *BRAF*-mutant RKO cells were resistant to inhibition of PI3K/AKT signaling by PI-103. In contrast to pharmaceutical approaches, the genetic *BRAF-*knockout inactivates B-Raf^V600E^ completely by definition. Thus, since we show a distinct decrease of AKT phosphorylation in RBW-1 cells, the genetic targeting alone might already represent the effect of a combined inhibition of both signaling pathways. Against this background, unspecific off-target effects might impact the unselective pharmaceutical approach, emphasizing the need for a conscientious molecular characterization of each compound. However, resistance towards PI-103 treatment in BRAF wild-type cells remains to some extent unexpected and might be explained by the multiple genetic defects reported in RKO, including a bi-allelic nonsense mutation of *NF1*[56].

### Confirmation of results in independent *BRAF*-knockout cells

Somatic cell gene targeting is known to provide a high degree of confidence [57,58] and additionally, genetic uniformity among our cell clones has been achieved by subcloning RKO-E1 from the parental cell line. However, during the course of recombination of the second *BRAF* allele, only one *BRAF*^(wt/-/-)^ clone was gained and verification of the results in further clones of each phenotype was desired [19].

**Figure 6:**
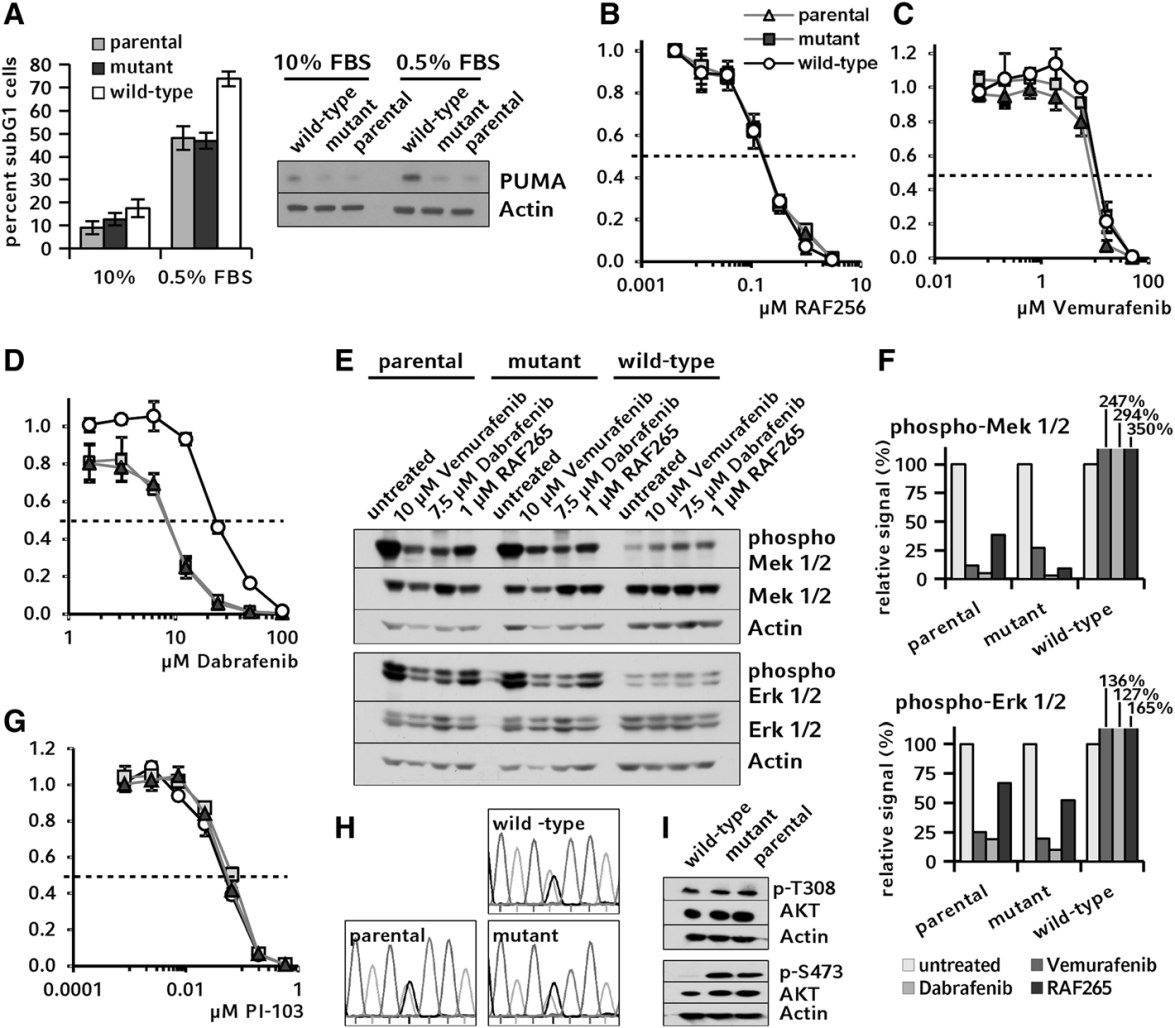
**Verification of results in independent *BRAF *knockout cells. A**: Cell death was assessed by determining the sub G1-fraction by flow cytometry and analyzed on a molecular level by Western blotting for PUMA. **B-D**: In chemosensitivity assays RAF265 and vemurafenib did not induce different responses, while with dabrafenib, the wild type clone showed a 2.6 fold higher IC_50_ value as compared to control clones. **E-F**: In Western blotting experiments and subsequent densitometry analysis, IC_50_ concentrations of dabrafenib showed a stronger effect on relative phosphorylation levels of Mek 1/2 and Erk 1/2 in parental and *BRAF*-mutant cells than RAF265 and vemurafenib. **G**: In proliferation assays, no differential responses were observed for PI-103. **H**: *PIK3CA* status was confirmed by sequencing. **I**: Phosphorylation of AKT was examined by Western blotting.

Therefore, we confirmed our data using a panel of similar RKO *BRAF*-knockout clones, which were established independently in a different lab and published during the course of our study [20]. Consistent with the findings from our cells, the *BRAF* wild-type clone from the complementary set of cells revealed the highest sub G1-fraction and strongest PUMA expression levels after withdrawal of serum as compared to the corresponding *BRAF-*mutant clones (Figure 6A). Similarly, no significant sensitivity differences were observed for the B-Raf^V600E^ inhibitors RAF265 and vemurafenib between *BRAF-*mutant and wild-type clones (Figure 6B and C). Dabrafenib selectively inhibited growth of cells containing mutant *BRAF* alleles at 3-fold lower IC_50_ as compared to *BRAF*-mutant clones (Figure 6D). Additionally, the relative phosphorylation levels of Mek 1/2 and Erk 1/2 were assessed by Western blotting in these cells. The relative phosphorylation was found to be more efficiently reduced by dabrafenib than by vemurafenib or RAF256 in *BRAF*-mutant cells on both Mek 1/2 and Erk 1/2 level, supporting the data obtained with our panel of corresponding cell clones (Figure 6E-F). However, while the wild-type clone of the confirmatory cell panel consistently showed the expected MAPK hyperactivation, the pattern among Mek 1/2 and Erk 1/2 levels differed markedly compared to our RBW-1 cells. As phosphorylation levels of these effectors show a complex temporal pattern, these differences are likely explainable by even slight variations in sample preparation [54].

Last, the unexpected resistance of RKO-derived *BRAF* wild-type cells towards inhibition of PI3K/AKT signaling was confirmed using the independent *BRAF-*knockout cell panel. As observed in our set of cells, no change of IC_50_ after PI-103 treatment was observed for the wild-type clone in the confirmatory cell set, while the *PIK3CA* phenotype was conserved and AKT phosphorylation was decreased under basal culture conditions (Figure 6G-I).

## Conclusions

Utilizing a *BRAF-*model of isogeneic cell lines, we provide evidence that V600E-mutant B-Raf confers independence of serum-derived growth factors and resistance to starvation-induced apoptosis, but not chemotherapy-induced apoptosis, indicating these traits to be main targets for B-Raf inhibitor therapy. Targeting of mutant *BRAF* alleles leads to a loss of these hallmarks of cancer. In contrast, B-Raf^V600E^ did not affect sensitivity towards conventional chemotherapeutic compounds such as mitomycin C, oxaliplatin, paclitaxel, etoposide, or 5-fluorouracil in our model. Also, no sensitivity was observed towards the therapeutic EGFR antibody cetuximab, although the EGF receptor was similarly expressed in all RKO-derived cell clones. Dissecting the effect of selective B-Raf inhibition, neither vemurafenib nor RAF265 induced proliferation differences among wild-type and mutant clones. In contrast, dabrafenib exhibited an obvious *BRAF* status-dependent inhibitory effect on cell proliferation. Together with the highly robust molecular effects of dabrafenib on phospho-Erk and phospho-Mek induction, this possibly indicates a high specificity of the compound. On the other hand, off-target effects could also have contributed, since all small molecule kinase inhibitors are multi-kinase inhibitors to some extent. Kinomic approaches to obtain detailed inhibition profiles appear as a promising tool for future studies to reveal the key differential modes of action between the utilized compounds.

## Methods

### Tissue culture

Cell culture reagents and antibiotics were purchased from PAA Laboratories (Pasching, Austria). HEK293 and RKO were purchased from ATCC (via LGC Standards GmbH, Wesel, Germany) and validated by DNA profiling at the German Biological Resource Center (DSMZ, Braunschweig, Germany). Additional RKO clones harboring deleted *BRAF* alleles were kindly provided by B. Vogelstein (Johns Hopkins University, Baltimore, MD) HEK293, RKO, and derivative cell clones were maintained at 37°C in a water-saturated atmosphere containing 5% CO_2_ in high glucose (4.5 g/L) DMEM supplemented with 100 units/mL penicillin, 100 mg/L streptomycin, and 10% FBS if not indicated differently.

### Somatic cell gene targeting

For somatic cell gene targeting, the AAV Helper-Free System (Agilent Technologies, Santa Clara, CA, USA) was used [18]. The chromosomally stable target cell line RKO shows slight aneuploidy leading to triplication at the *BRAF* locus (dup (7) (q21q36)) [13]. In order to target two *BRAF* alleles serially, two AAV targeting constructs were cloned containing either hygromycin or neomycin resistance. The resistance cassette was flanked by sequences homologous to regions flanking *BRAF* exon 15 (Figure 1A). These homology arms were amplified by PCR using primer LHA_FW_NotI (atacatac-GCGGCCGC-tgactggagtgaaaggtttg) with LHA_RV_linkA (GCTCCAGCTTTTGTTCCCTTTAG-cattttcctatcagagcaagc), or RHA_FW_linkB (CGCCCTATAGTGAGTCGTATTAC-gtggatggtaagaattgagg) with RHA_RV_NotI (atacatac-GCGGCCGC-catgagtggcctgtgattc), respectively. Preparation of AAV particles was done according to Kohli *et al*[18].

After a limiting dilution of RKO cells, the single clone RKO-E1 was infected with AAV containing the *hyg* resistance gene and seeded in a limiting dilution. After three weeks of incubation with 2.0 g/L hygromycin B, single colonies were screened with two primer pairs: LHA-upstream-FW (agggacatggataaataggcttg) combined with CMV-5′-RV (tagggcgcgataacttcgta) and RHA-downstream-RV (agcaggccagtcaactcct) in combination with BGHpA-3′-FW (ccgaggagcaggactgaata). In order to verify the successful recombination, a genomic region of approximately 300 bp was amplified with exon 15 flanking primers BRAF-E15-300-FW (gccccaaaaatcttaaaagca) and BRAF-E15-300-RV (ctgatgggacccactccat) and was subsequently analyzed by DNA sequencing using BRAF-E15-300-seq (ttattgactctaagaggaaagatgaa).

From a clone of the desired *BRAF* genotype (oncogenic/wild-type/deleted) the knockout cell line RBOW (RKO-derived clone *BRAF*^onc/wt/-^) was established. RBOW cells were infected with AAV particles mediating neomycin resistance, diluted and incubated with 4.5 g/L G418 sulphate. For PCR screening of the single colonies, LHA-upstream-FW was combined with Neo-5′-RV (gttgtgcccagtcatagccg) and RHA-downstream-RV was combined with Neo-3′-FW (tcgccttcttgaagagttct). Positive clones were verified as above. The knockout clones RBO-1, RBO-2 (RKO-derived clone *BRAF*^onc/-/-^ 1 and 2) and RBW-1 (RKO-derived clone *BRAF*^wt/-/-^ 1) were further expanded.

### Western blotting

Western blot samples were prepared with phospho-protein lysis buffer. Blocking of the membranes was done with 5% BSA in TBS-T prior to the detection of phospho-proteins, or else with 5% skim milk powder in TBS-T. Antibodies against B-Raf, pan Mek 1/2, phospho-Mek 1/2, pan Erk 1/2, phospho-Erk 1/2, caspase 3, and p53 (item numbers 9434, 9122, 9121, 4695, 4370, 9665, and 2524) were purchased from Cell Signaling Technologies (via New England Biolabs GmbH, Frankfurt am Main, Germany). For detection of EGFR and PUMA item numbers sc-03 and sc-374223 from Santa Cruz Biotechnology (Santa Cruz, CA, USA) were used. Actin was detected with actin monoclonal antibody from MP Biomedicals (Solon, OH, USA). Densitometry was done with ImageJ software by Wayne Rasband.

### Staining of senescence-associated β-galactosidase activity

Cellular senescence was detected by staining of senescence-associated β-galactosidase activity at pH 6.0 [25]. To facilitate detection of positive blue cells, the cells were counterstained with 0.1% rosinduline in 1% acetic acid. Cells were air-dried and quantified by bright field microscopy.

### Flow cytometry

Flow cytometry was performed either on a BD FACSCalibur (BD Biosciences, San Jose, CA, USA) or an Accuri C6 (BD Bioscience) device. Data analysis was done using Flowing Software by Perttu Terho and CFlow Plus (BD Bioscience), respectively.

### Proliferation and chemosensitivity assays

For proliferation assays, 10^5^ cells were seeded in 6-well plates in triplicates and incubated for the indicated time period. Every 24 hours, triplicates were trypsinized and diluted according to the expected cell yield estimated in advance by phase-contrast microscopy. For each replicate two aliquots of 10 μL were taken and counted in a 3x3 square hemacytometer. For each triplicate of sample at each time point, standard error of the mean (SEM) was calculated.

Chemosensitivity assays were performed using standard SYBR green cell proliferation assays over a broad range of concentrations (covering 100% to 0% survival), as described previously [59,60]. Briefly, cells (1,500–1,800 per well) were plated in 96-well plates, allowed to adhere, and subsequently treated. After seven days, the cells were washed and lyzed in 100 µL of deionized water, and 0.2% SYBR green I (Lonza Group Ltd., Basel, Switzerland) was added. Fluorescence was measured (Cytofluor Series 4000, Applied Biosystems, Darmstadt, Germany) and growth inhibition calculated as compared to the untreated control samples. At least three independent experiments were performed per agent, with each data point reflecting triplicate wells. Error bars represent standard error of the mean (SEM) from three experiments.

## Competing interests

The authors declare no competing interests.

## Authors’ contributions

Conception and design: BH, EG, FTK; acquisition of data: BH, AZ, MM; analysis and interpretation of data: BH, EG, FTK; writing and revision of the manuscript: BH, EG, AZ, FTK. All authors read and approved the final manuscript.
